# Social interest in publications on bruxism: an altmetric analysis

**DOI:** 10.1590/1807-3107bor-2025.vol39.113

**Published:** 2025-11-07

**Authors:** Aurélio de Oliveira ROCHA, Lucas Menezes dos ANJOS, Fernanda Pretto ZATT, Pablo Silveira SANTOS, Filipe Colombo VITALI, Bruno HENRIQUES, Mariane CARDOSO

**Affiliations:** (a) Universidade Federal de Santa Catarina – UFSC, Department of Dentistry, Florianopolis, SC, Brazil.; (b) Universidade Federal de Santa Catarina – UFSC, Department of Mechanical Engineering, Florianopolis, SC, Brazil.

**Keywords:** Altmetrics, Dentistry, Dental Occlusion, Bruxism

## Abstract

Metrics derived from online social platforms indicate current interest in a specific topic. This study aimed to analyze the characteristics and social interest in scientific publications on bruxism using an altmetric analysis. A search was conducted in August 2024 in the Dimensions database. The following data were extracted: altmetric attention score (AAS), citations, year, language, access type, study design, topic (general objective and age group), journal, country, institution, and authors. VOSviewer was used to generate collaborative networks, whereas Google Trends was consulted to assess public interest in bruxism-related research. A Spearman correlation analysis was performed to establish the relationship between AAS and citations. A total of 196 studies published between 1992 and 2024 were included. The most widely accessed study had an AAS of 393. Significant interest in bruxism was observed among Mendeley, news outlets, and X users. Most studies were observational (n = 99), addressing the etiologic factors of bruxism (n = 75) and focusing primarily on the adult population (n = 148). The Journal of Oral Rehabilitation stood out as the most relevant journal (n = 36). Most articles were published in Brazil (n = 40), and Lobbezoo was the most frequent author (n = 27). VOSviewer revealed significant collaborations among authors. Most studies were not openly accessible (n = 105). A very weak positive correlation (r = 0.042) was observed between AAS and the number of citations. This study highlighted a social interest in scientific publications on bruxism, particularly those addressing etiologic factors.

## Introduction

Bruxism is characterized by repetitive jaw muscle activity, represented by the habit of clenching or grinding teeth and/or by contracting or pushing the jaw.^1^ This condition can occur during sleep (referred to as sleep bruxism) or while awake (referred to as awake bruxism).^2^ The etiology of bruxism is multifactorial and not fully understood, which is why it sparks significant interest among researchers.^3^ Psychological and respiratory factors during sleep are commonly associated with the onset and progression of bruxism.^4^ A global systematic review observed that one in four people has bruxism.^5^ Various complications are associated with both sleep and awake bruxism, such as muscle pain, temporomandibular joint pain, difficulty sleeping, and irreversible dental wear, which can range from mild to severe.^6^ These conditions foster scientific production on the topic and motivate the search for information on the internet and social media. Several bibliometric analyses have been published on bruxism. Nevertheless, no altmetric analysis has been conducted.^7-10^

The dissemination of scientific knowledge typically depends on the publication of articles, and the impact of these publications is often measured by the number of citations they receive.^11^ Citation performance, however, often requires a prolonged period to generate an impact on visibility. Therefore, altmetrics have gained significant attention in the academic community, as they can measure immediate scientific interest in an article or topic based on its sharing on social media and online platforms.^12^ The altmetric attention score (AAS), the most prominent altmetric tool, measures the impact of social web activity on publications and has shown potential as a real-time indicator of attention in health research through news, blogs, and social media platforms.^13^ The AAS serves as an indicator designed to measure the level of online attention a publication has received. The AAS is calculated using a weighted algorithm that considers various factors, such as the volume of mentions and the influence of the sources. The AAS is always represented as an integer, with 1 being the minimum score in this analysis.^13^

With approximately five billion users worldwide, social media and online platforms facilitate the swift exchange of information and content. This extensive interaction between researchers and clinical dentists can indicate the current and genuine interest in a particular topic or field of study.^14^ Citation-based metrics in academic research can take years to demonstrate the relevance and interest in a scientific subject. In contrast, altmetrics capture immediate and contemporary interest within a field, potentially guiding new research and future studies in a shorter time frame compared to traditional citation-based metrics.^15^

In dentistry, altmetrics are an emerging analytical approach often associated with bibliometric studies. Because of its relevance and uniqueness, this approach has been gaining increasing prominence and importance.^12,16^ Therefore, the aim of the present study was to assess the reach and research interest in bruxism across online media platforms using an altmetric analysis.

## Methods

### Information sources and search strategy

An electronic search was conducted in August 2024 using the Dimensions database (dimensions.ai). The search strategy combined MeSH (Medical Subject Headings) terms and free-text keywords using the Boolean operator “OR”: (Bruxism* OR “Clenching” OR “Tooth Grinding” OR “Teeth Grinding” OR “Grinding of the Teeth” OR “Grinding of Teeth” OR “Grinding of Tooth”). Wildcard operators (truncation), such as “clench*” and “grind*”, were tested to evaluate the sensitivity of the search. The inclusion of these operators did not alter the retrieved records. Therefore, the original search terms were maintained. The “Dentistry” filter in Dimensions was applied to limit the results to the dental field. Additionally, the global popularity of the search term “bruxism” was explored using Google Trends.

### Eligibility criteria

A comprehensive search strategy was employed to identify studies that investigate bruxism in the context of dentistry. No restrictions were applied in terms of language, geographic location, year of publication, or studied population. Studies that did not report an AAS in Dimensions or did not assess bruxism were excluded. Therefore, only studies related to bruxism with an AAS of at least one (1) were included.

### Studies selection and data collection

The documents retrieved from Dimensions were organized in descending order based on their AAS and screened by title and abstract, with full-text reading performed when necessary. Two reviewers (AOR and LMA) independently conducted the selection process. Subsequently, both reviewers cross-checked the included studies to identify any discrepancies, which were resolved through consensus with a third reviewer (MC). The screening was performed manually, and the selected studies were exported to a Microsoft Excel spreadsheet (Microsoft Corporation, Redmond, WA, USA) for data extraction.

After the selection of the articles, the following characteristics were extracted: AAS, number of citations, year of publication, journal, access type, study design, topic, country, author, and institution. Based on the publication type category [Publication Type] of MeSH (Medical Subject Headings), the study designs were classified as follows: systematic review, scoping review, literature review (narrative, integrative, or critical), consensus, observational study (prospective, retrospective, cross-sectional, case-control, or cohort), clinical trial (randomized or non-randomized), laboratory study (*in vitro* or *in vivo*), and case series or reports. Studies that did not fit into these categories were grouped as “others.”^17^

The main research topic was defined based on the primary outcome of each study, which was determined by analyzing the main objective stated in the main text. In cases in which the primary outcome was not explicitly stated, the classification was based on the methodological focus and central research question. The studies were categorized into the following research topics: bruxism occurrence (prevalence or incidence assessment), etiology (risk or associated factors), diagnosis of bruxism, management of bruxism, clinical consequences of bruxism, and scientific status (analysis of existing literature related to a bruxism-related topic). Additionally, the age range of the participants was assessed and classified as either child/adolescent (0 to 17 years) or adult (18 years or older).^18^

### Collaboration and performance analysis

The Visualization of Similarities Viewer software (VOSviewer, version 1.6.17.0, Netherlands) was used to create graphical representations of bibliometric networks based on co-authorship among author groups and countries. The documents obtained from Dimensions were exported as a text file (.txt) to ensure compatibility with VOSviewer. For the author-level co-authorship analysis, only authors with a minimum of four publications were included, and author names were standardized to consolidate spelling variations. All identified countries were included in the country-level co-authorship analysis, given the Mesh small number of countries identified. The association strength method (default normalization in VOSviewer) was applied in both analyses. In the resulting maps, each cluster of collaborating authors or countries is displayed in a distinct color, with larger circles representing higher publication frequency. Proximity between circles reflects the strength of co-authorship links, and thicker lines indicate stronger collaborative relationships.

### Statistical analysis

Data analysis was performed using the *Statistical Package for the Social Sciences* (SPSS) software, version 24.0 (IBM Corp., Armonk, USA). Initially, the Kolmogorov-Smirnov test was employed to determine whether the data exhibited a normal distribution. Considering the non-normal distribution of the data (p < 0.05), Spearman’s rank correlation coefficient was utilized to assess the correlation between the AAS, the number of citations, and the year of publication.

## Results

**Figure 1:**
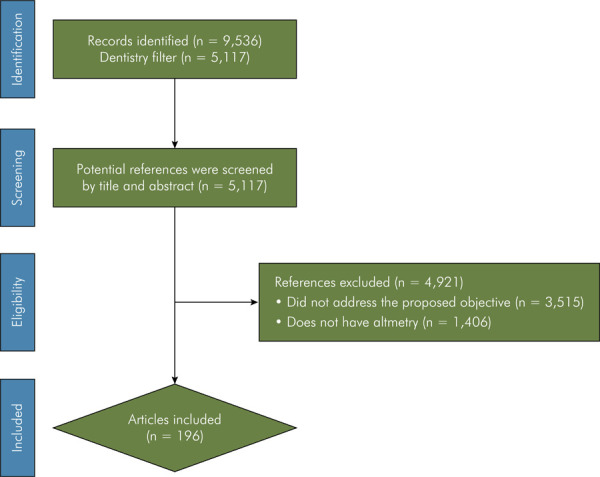
Flowchart of the study selection process.

The initial search in Dimensions retrieved 9,536 documents. By applying the “Dentistry” filter, 5,117 documents were identified. Among the retrieved articles, 196 studies demonstrated quantifiable altmetric performance. All studies with altmetric data were included for analysis. Figure 1 details the article selection process.

The selected articles had an AAS ranging from 1 to 393. The article with the highest social media interest (AAS = 393) was “Temporomandibular Disorders and Bruxism Outbreak as a Possible Factor of Orofacial Pain Worsening during the COVID-19 Pandemic - Concomitant Research in Two Countries.”^19^ This article has an altmetric score of 393, which includes news (51), blogs (2), X (7), Facebook (4), video (1), and Mendeley (242). Table displays the 10 studies with the highest altmetric performance and citations in Dimensions.

**Table:** Top 10 articles on bruxism with the highest altmetric performance.

AAS	Articles	Citations	Mentioned by
**393**	Emodi-Perlman A, et al. Temporomandibular disorders and bruxism outbreak as a possible factor of orofacial pain worsening during the COVID-19 pandemic-concomitant research in two countries. J Clin Med. 2020 Oct 12;9(10):3250.	102	242 mendeley
			51 news outlets
			7 X users
			4 Facebook pages
			2 blogs
			1 YouTube creator
**382**	Kardeş E, Kardeş S. Google searches for bruxism, teeth grinding, and teeth clenching during the COVID-19 pandemic. J Orofac Orthop. 2022 Nov;83(6):1-6.	12	50 news outlets
			45 Mendeley
			4 X users
			1 policy source
**240**	Alkhatatbeh MJ, et al. Self-reported sleep bruxism is associated with vitamin D deficiency and low dietary calcium intake: a case-control study. BMC Oral Health. 2021 Jan 7;21(1):21.	13	78 Mendeley
			33 X users
			30 news outlets
			7 YouTube creators
**210**	van der Meer HA, et al. The association between headaches and temporomandibular disorders is confounded by bruxism and somatic symptoms. Clin J Pain. 2017 Sep;33(9):835-843.	37	142 Mendeley
			25 news outlets
			7 X users

**205**	Martynowicz H, et al. Evaluation of relationship between sleep bruxism and headache impact test-6 (hit-6) scores: a polysomnographic study. Front Neurol. 2019 May 14;10:487.	21	41 Mendeley
			29 news outlets

**197**	Tokiwa O, et al. Relationship of tooth grinding pattern during sleep bruxism and dental status. Cranio. 2008 Oct;26(4):287-93.	36	70 Mendeley
			24 news outlets
			1 Wikipedia page
**139**	Khoury S, et al. sleep bruxism-tooth grinding prevalence, characteristics and familial aggregation: a large cross-sectional survey and polysomnographic validation. Sleep. 2016 Nov 1;39(11):2049-2056.	69	162 Mendeley
			14 news outlets
			1 blog
			1 X user
			1 Facebook page
			1 Wikipedia page
**129**	Entezami S, et al. Tooth wear and socioeconomic status in childhood and adulthood: Findings from a systematic review and meta-analysis of observational studies. J Dent. 2021 Dec;115:103827.	10	49 Mendeley
			15 news outlets
			8 X users
			2 blogs
**127**	Bertazzo-Silveira E, et al. Association between sleep bruxism and alcohol, caffeine, tobacco, and drug abuse: a systematic review. J Am Dent Assoc. 2016 Nov;147(11):859-866.e4.	74	159 Mendeley
			45 X users
			11 Facebook pages
			10 news outlets
			2 blogs
			2 Google+ users
			2 YouTube creators
**100**	Yap AU, Chua AP. Sleep bruxism: current knowledge and contemporary management. J Conserv Dent. 2016 Sep-Oct;19(5):383-9.	100	261 Mendeley
			12 news outlets
			9 X users
			1 patent
			2 Facebook pages

The selected articles received a total of 12,916 citations in Dimensions. The most cited article, with 835 citations, was “Bruxism defined and graded: an international consensus.”^20^According to Spearman’s correlation, a very weak positive correlation (r = 0.042) was observed between the AAS and the number of citations. A very weak positive correlation was also found between the AAS and the year of publication (r = 0.108).

**Figure 2:**
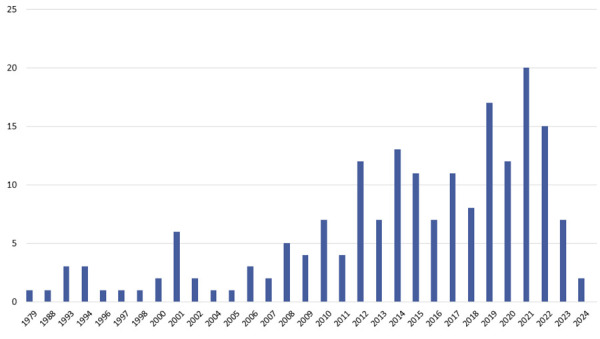
Distribution of the number of publications by year.

The oldest article included in this study was published in 1979: “Bruxing and non-bruxing children: a comparison of their personality traits,”^21^ while the most recent articles were published in 2024 (n = 2). A noticeable increase in the number of publications was observed over the past five years (n = 73). The distribution of publications per year is shown in Figure 2.

All articles were published in English (100%). Regarding access type, only 46.4% of the publications were classified as open access (n = 91). The average AAS for open-access studies was 40.30, while closed-access articles had an average of 18.06. In terms of citations, open-access articles averaged 63.7 citations, while closed-access articles averaged 67.74 citations.

Regarding study design, most were observational (n = 99), followed by literature reviews (n = 31), systematic reviews (n = 29), clinical trials (n = 24), case reports (n=4), consensus studies (n = 3), laboratory studies (n = 2), and scoping reviews (n = 1). Three studies did not fit into the categories and were grouped as “others” (pilot studies). According to the primary objective of the studies, there was a predominance of research focused on etiologic factors or those associated with the development of bruxism (n = 75), management of bruxism (n = 53), oral consequences resulting from bruxism (n = 29), diagnosis of bruxism (n = 16), prevalence (n = 12), and scientific status (n = 11). In terms of age, most studies involved adult patients (n = 148), followed by children and/or adolescents (n = 41). Seven studies were conducted for both children/adolescents and adults.

A total of 94 journals contributed to the studies on bruxism with altmetric performance. The journal with the largest number of publications was the Journal of Oral Rehabilitation (n = 36), followed by the British Dental Journal (n = 8), Journal of Clinical Medicine, Cranio, and the Journal of the American Dental Association (n = 6 each). Journals featuring highlighted fonts and enclosed in circles with more saturated tones of red exhibited the highest prominence.

**Figure 3:**
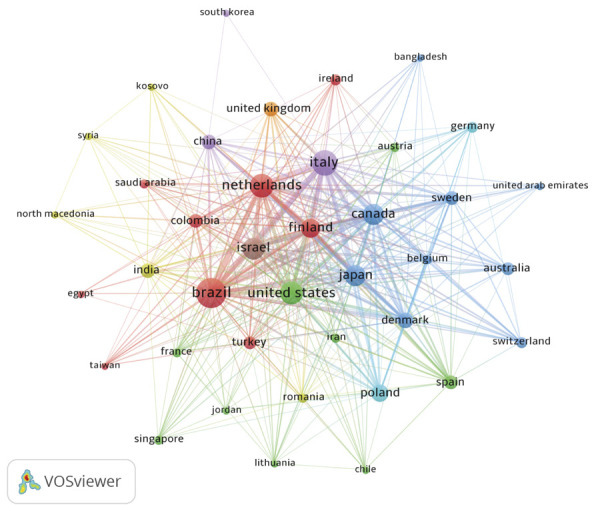
Global distribution and collaboration between contributing countries.

A total of 39 countries contributed to the broader social reach of bruxism research. The leading country was Brazil (n=40), followed by Italy (n=26) and the Netherlands (n = 23). Figure 3 illustrates the identified countries and their collaborations.

**Figure 4:**
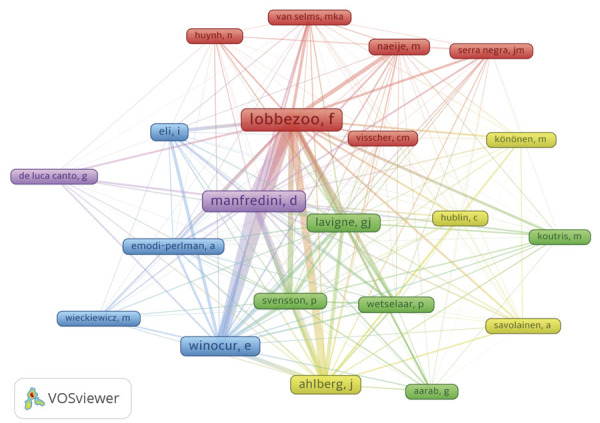
Most prominent authors and interaction between author groups.

In total, 316 institutions were associated with publications on bruxism that showed altmetric performance. The most prominent institution was the Academic Center for Dentistry Amsterdam (n = 21), followed by Tel Aviv University (n = 17) and University of Padua (n = 15). Among the contributing authors (n=754), Lobbezoo F (n = 27) was identified as the most prominent , followed by Manfredini D (n = 19) and Winocur E (n = 14). The collaboration network and the main author groups are illustrated in Figure 4 (more than four occurrences).

Based on Google Trends data, the global popularity of “bruxism” research has remained steady over the past 20 years. Conversely, Romania, Australia, New Zealand, the United States, and Ireland exhibited significant research interest. The most common related research topics included “human dentition,” “masseter,” and “apnea.” Users searching for “bruxism” also explored related queries. The most popular search queries were “bruxism botox,” “mouth guard for bruxism,” and “bruxism splint.”

## Discussion

Bruxism poses significant challenges both in diagnosis and treatment.^1-3^ Understanding the genuine interest in this topic is essential for its scientific development. Therefore, the aim of this study was to investigate the reach and social interest in bruxism research through an altmetric analysis. Studies with notable altmetric performance were published in recent years, primarily with an observational design, mostly focused on determining the etiology and factors associated with bruxism in adult patients. These studies largely originated from Brazil and were mainly published in the Journal of Oral Rehabilitation.

In 2018, Digital Science & Research Solutions introduced Dimensions, an online academic platform designed to provide a unique perspective on research developments by displaying altmetric data. The platform considers various indicators, such as awards, journal and book publications, social media mentions, academic citations, clinical trials, and commercial patents. The choice to use this database was based on its broad analysis of the online environment, providing robust information. Additionally, this database has been used in other altmetric studies in the literature.^12,22,23^

The AAS is a metric used to measure the attention and online engagement that scientific work (such as an article, book, or dataset) receives across various platforms and media.^24^ Unlike traditional citation metrics (such as impact factor or citation count), which primarily focus on academic citations, the AAS aggregates attention from various non-academic sources, such as social media, news outlets, policy documents, government reports, reference managers, academic forums, and Wikipedia.^12^ The article with the highest AAS for bruxism aimed to assess the effect of the COVID-19 pandemic on the potential prevalence and worsening of temporomandibular disorder and bruxism symptoms among selected individuals from two culturally different countries: Israel and Poland.^19^ The pandemic was a period of global stress, and this condition has the potential to induce the onset or exacerbation of bruxism cases. This fact justifies the social interest in the article.

The AAS is calculated based on mentions of a publication across various online sources. These sources are assigned different weights depending on their perceived influence; for instance, news outlets are generally considered to have a greater influence than social media mentions. While it is well understood that news stories exert the most significant influence on the score - followed by blogs, public policy documents, Wikipedia entries, patents, and social media platforms such as X (formerly Twitter), Facebook, and YouTube - the exact weighting algorithm used by Altmetric.com is proprietary and not publicly disclosed.^13,15,26^ Consequently, the interpretation of AAS values should be approached with caution, as the scoring process may prioritize certain types of attention in ways that remain unclear. In the present study, publications on bruxism received substantial attention from Mendeley readers, reflecting academic interest, as well as from news outlets and social media users, indicating broader public engagement.

The AAS is updated in real time, providing a dynamic view of the attention that research is receiving.^25^ This is evident by observing that most of the studies with altmetric performance were published in recent years, with a peak in 2021 (n=20). Altmetrics demonstrate immediate attention to a given study, unlike citation metrics, which require a longer period for an article to be cited sufficiently.^22^ This distinction was evident in Spearman’s correlation, which showed a weak positive correlation between the AAS and the number of citations. Articles with higher citation counts were identified by bibliometric reviews as older studies.^11,27^

A greater trend in altmetric performance was observed for open-access studies, unlike citation performance, in which the number of citations was similar for both open- and closed-access studies. This could be explained by the fact that citations are often generated by researchers who have easier governmental access to open-access studies. In contrast, altmetrics can be generated by the general population, who may not have access to closed-access studies due to the limitations imposed by higher education institutions.^12,28^This highlights the importance of open access publications, as they ensure equal access for all audiences and promote evidence-based knowledge across different social groups.

Observational studies are important sources of scientific evidence.^5^ These studies are well-suited for assessing and identifying etiologic factors associated with a disease. Accordingly, greater social interest in bruxism was observed for observational studies that investigated etiologic or associated factors. Bruxism can have a complex and multifactorial etiology, and its treatment depends on the clinical identification and removal of the causative factors.^20^ Furthermore, literature reviews were another highly sought-after study design, as this type of research provides broad and detailed information on a given condition, which can explain the interest in this design. On the other hand, studies with a higher level of scientific evidence, such as clinical trials and systematic reviews, were less sought after, indicating that social interest needs a stronger scientific basis.

According to Google Trends, global interest in the search term “bruxism” has remained consistent over the past two decades. Individuals who searched for “bruxism” also searched for terms such as “bruxism botox,” “mouth guard for bruxism,” and “bruxism splint.” These terms align with the second most frequently investigated topic among the studies with altmetric data, which was related to the management of bruxism. This topic was primarily represented by clinical trials and systematic reviews, thereby increasing the level of scientific evidence within this category.

The Journal of Oral Rehabilitation had the highest number of publications, and other studies based on metrics also identified it as the leading journal for publications on bruxism.^7,9^ The Journal of Oral Rehabilitation is a prestigious dental journal dedicated to the publication of research related to oral rehabilitation and applied oral physiology. It covers all aspects of diagnosis and clinical management necessary to restore harmonious subjective and objective oral function.

Altmetric analysis has gained prominence over traditional metrics (such as citations) within the scientific domain. Its main contribution lies in providing a broader, more immediate, and socially contextualized view of the impact of scientific research, thereby indicating the societal interest in a given scientific approach.^12^ Altmetrics allow for the identification of how and where research is being discussed outside the academic sphere, revealing the immediate interest an article generates after its publication. Altmetric performance encourages researchers to engage with broader audiences who show interest in their findings.^15^ By highlighting which articles are being widely discussed in real time, altmetrics help identify public interest topics before they become academically consolidated, potentially influencing and shaping future scientific research.^23^ In the scientific context, this study demonstrated that outside academia, there is significant societal interest in observational studies investigating the etiologic factors of bruxism, particularly in the adult population.

The present study has several important strengths, including its focus on a significant research topic and the use of a contemporary methodological approach that incorporates altmetric indicators, providing new insights into how scientific literature on bruxism circulates across online platforms. Several limitations, however, warrant discussion. First, relying on a single altmetric platform (Dimensions) may have affected the comprehensiveness of the results. Although Dimensions integrates multiple sources, it does not capture all types of online attention. This could result in a partial or skewed view of how certain articles were received and shared online. Moreover, the proprietary nature of altmetric algorithms restricts transparency regarding how scores are calculated and why certain articles may be prioritized over others. Consequently, some types of content or publication venues might be disproportionately emphasized. To address these issues, we recommend that future studies consider incorporating multiple altmetric platforms for cross-verification and a more complete picture of public and scientific engagement. Additionally, the Google Trends analysis was confined to a single search term (“bruxism”). While this ensured consistency with the bibliometric strategy, it may have overlooked public interest related to lay expressions such as “teeth grinding” or “tooth clenching.” Another limitation is the classification of studies based on their primary outcome, which may also introduce subjectivity, particularly when the authors do not clearly define the primary objective. In such cases, classification relied on methodological focus and thematic relevance, which may not always reflect the authors’ original intent. Finally, the study protocol was not prospectively registered, which may limit reproducibility. We encourage future altmetric studies to adopt predefined classification criteria and register their protocols on platforms such as the Open Science Framework (OSF) to enhance transparency and methodological rigor.

## Conclusion

This study highlighted significant social interest in scientific publications related to bruxism, particularly those investigating etiologic factors in adult populations, with a substantial number published in the Journal of Oral Rehabilitation. Although most studies originated from Brazil, the most notable author identified was Lobbezoo F. Interestingly, the articles with the highest altmetric performance were predominantly published under closed access. A very weak correlation was found between the AAS and the number of citations, suggesting limited overlap between social media attention and academic impact.

## Data Availability

The authors declare that all data generated or analyzed during this study are included in this published article.
